# Editorial: Functionalized Inorganic Semiconductor Nanomaterials: Characterization, Properties, and Applications

**DOI:** 10.3389/fchem.2020.616728

**Published:** 2020-11-11

**Authors:** Kezhen Qi, Rengaraj Selvaraj, Liwei Wang

**Affiliations:** ^1^Institute of Catalysis for Energy and Environment, College of Chemistry and Chemical Engineering, Shenyang Normal University, Shenyang, China; ^2^Department of Chemistry, College of Science, Sultan Qaboos University, Muscat, Oman; ^3^School of Marine Sciences, Guangxi University, Nanning, China

**Keywords:** functionalized inorganic semiconductors, nanocrystals, photocatalysis, quantum dots, nanorods, biosensors

Nanotechnology involves studying and working with matter on a nanoscale, which provides the ability to work at the atomic level and molecular level to create large structures with fundamentally new molecular organization. The field of nanotechnology presents an exciting and rapid expansion of research area that crosses the barriers among physics, chemistry, biology, life, and engineering sciences. Nanostructure materials generally called nanomaterials are the materials having at least one dimension between 1 and 100 nm. These materials exhibit novel and significantly improved physical, chemical, and biological properties, phenomena, and processes due to their nanoscale size (Liu et al., 2019; Marzouqi et al., 2019; Qi et al., 2019a,b).

The functionalized semiconductor based nanomaterials with different morphologies and compositions have been successfully applied for numerous applications (Wang et al., 2018; Qi et al., 2020b). Depending on their size and shape, the physical, chemical, electrical, and optical properties of the functionalized nanomaterials are different as compared to their bulk structures (Ruqaishy et al., 2018; Qi et al., 2020a). Because of their small size, the nanomaterials have large surface area and high surface/volume ratio (Al-Fahdi et al., 2019). This high surface/volume ratio is one of the reasons that nanomaterials have superior chemical and physical properties such as large surface energy, reactivity, solubility and low melting point as compared to their bulk counter-parts (Yu et al., 2007; Qi et al., 2018; Hayat et al., 2019). Decreasing size of the material causes an increase in surface area. The functional properties of nanomaterials depend mainly on their unique structures, which can be classified into three levels namely, the microscale, mesoscale, and particle scale.

In this Research Topic, we present a collection of original research and review articles focussing on different aspects of functionalized inorganic semiconductor nanomaterials, including modified Ag_3_PO_4_ semiconductors for improved photocatalytic performance (Liu Q. et al.), hydrogen production and pollutant degradation by functionalizing g-C_3_N_4_ with SnO_2_ (Zada et al.), SiO_2_ coated ZnO nanorod arrays for UV-durable super hydrophobicity (Li et al.), synthesis of micro/nanoscale LiFePO_4_/Graphene for lithium-ion batteries (Liu S. et al.), facile and efficient fabrication of bandgap tunable carbon Quantum Dots (Jia et al.), Calcium-phosphate lipid system for potential dental application (Zhu et al.), VOC gas sensors based on molybdenum oxide (Wang J. et al.; Wang S. et al.), metal oxides—based semiconductors for biosensors (Serban and Enesca), ZnO nanorods for detecting reducing gases (Aljaafari et al.), ZnO nanomaterials: Current advancements in antibacterial mechanisms (Jiang et al.), quantum dot sensor for the detection of calcium ions (Ghosh et al.), mixed electronic and ionic charge carrier localization and transport (Romero et al.), and mesoporous silica nanoparticles for controlled release of antimicrobials for stone preventive conservation (Presentato et al.). We have also highlighted that how state of the art theoretical and experimental approaches are leading to better understanding of semiconducting materials and improved design of novel functional semiconducting materials.

The contribution of Qian et al. concerns an effect of ammonium phosphate—modified Ag_3_PO_4_ on photocatalytic performance. The authors demonstrated a novel one-pot surface modification route by using ammonium phosphate solutions to improve the photocatalytic performance of Ag_3_PO_4_. It was found that ammonium phosphate played multiple promotion roles in favoring the formation of metallic Ag nanoparticles and providing the negative electrostatic field on the surface of Ag_3_PO_4_ photocatalysts, which consequently promoted the separation efficiency of photoinduced electron-hole pairs, enhanced selective adsorption of cationic dye, and increased concentration of reactive oxygen species. This work provides an alternative route to boost the photocatalytic activity of Ag_3_PO_4_.

The work of Zada et al. investigated the functionalization of g-C_3_N_4_ with SnO_2_ for the photocatalytic hydrogen production and degradation of pollutants. This work is emphasized to overcome energy crises and environmental pollution. The authors synthesized g-C_3_N_4_ nanosheets and coupled them with SnO_2_ nanoparticles. The enhanced photoactivities were attributed to the better charge separation as the excited electrons thermodynamically transferred from g-C_3_N_4_ to SnO_2_ as had been confirmed from photoluminescence spectra, steady state surface photovoltage spectroscopic measurement, and formed hydroxyl radicals. It is believed that this work would provide a feasible route to synthesize photocatalysts for improved energy production and environmental purification.

The contribution of Li et al. developed vertically aligned ZnO nanorod arrays with large area through chemical hydrothermal process. Ultra-thin SiO_2_ shell film was deposited on ZnO nanorod arrays through PLD, and subsequently modified by stearic acid. This SiO_2_/ZnO/glass structure exhibited well UV-durable super hydrophobicity and high transmittance. These properties have important applications in solar cells.

Liu S. et al. prepared LiFePO_4_/graphene composites by packing LiFePO_4_ nanoparticles in the micron graphene sheets by one-step microwave heating technique. The introduction of graphene did not affect the structure of LiFePO_4_ as the nanoparticles were surrounded by the graphene sheets and the micron structure guarded the stability of the material. The electrochemical analysis reveal that the LiFePO_4_/graphene composites have excellent high-rate performance and cycling life. The outstanding electrochemical performance, as well as the fast and efficient method, make this technology commercially viable.

Jia et al. developed a facile, fast, and green method to prepare bandgap tunable CQDs solely from anthracite. The bandgap change of the as-prepared CQDs could be achieved by simply controlling the concentration of H_2_O_2_. The morphology, size and PL properties of the as-prepared CQDs indicated that the blue luminescence might be originated from the intrinsic emission, but the yellow and green luminescence might be originated from the extrinsic emission due to the new energy states created by the oxygen-containing functional groups inside the band gap of CQDs. This novel strategy for fabricating optically tunable CQDs from coal is highly promising for the high-end application of coal.

The work of Zhu et al. investigates the calcium-phosphate lipid system for potential dental application. Aljaafari research group successfully synthesized ZnO nanorods using a domestic microwave-assisted solution method and showed a smooth surface morphology and wurtzite hexagonal structure. They concluded that the fabricated ZnO NRs using the microwave method was very sensitive to CH_4_ and CO, where the sensitivity toward these two gases was very high compared to H_2_ gas. The smooth surface of nanorods could also be used as a high operating temperature sensor.

Jiang et al. (2020) studied the antibacterial properties, mechanism, and application prospects of ZnO nanoparticles. The excellent biocompatibility, photochemical stability, and other characteristics of ZnO nanoparticles make it suitable for antibacterial activity. They concluded that doping with other metals or non-metallic materials to enhance the selectivity for pathogenic microorganisms and reduce the toxic effect of tissue cells might exert more extensive biomedical potentials for ZnO nanoparticles.

Ghosh et al. developed a simple optical aptasensor for the detection of calcium ions. The sensor had been found to have high specificity for calcium ions in comparison to other metal ions like sodium, magnesium, and potassium. The molecular apta-beacons also demonstrated successful endocytosis and FRET-based calcium ion detection in osteocyte cells when conjugated with a cell-penetrating peptide (DSS).

We hope this Research Topic will attract readers, providing novel literature insights, synergistic research ideas, and enthusiasm in research and studies.

## Author Contributions

All authors listed have made a substantial, direct and intellectual contribution to the work, and approved it for publication.

## Conflict of Interest

The authors declare that the research was conducted in the absence of any commercial or financial relationships that could be construed as a potential conflict of interest.
